# Antimalarial and cytotoxic drugs on COVID-19 and the cardiovascular burden: Literature review and lessons to be learned

**DOI:** 10.1177/1708538120941635

**Published:** 2020-07-21

**Authors:** Sherif Sultan, Yogesh Acharya

**Affiliations:** 1 Western Vascular Institute, Department of Vascular and Endovascular Surgery, University Hospital Galway, National University of Ireland, Galway, Ireland; 2 Department of Vascular Surgery and Endovascular Surgery, Galway Clinic, Doughiska, Ireland

**Keywords:** COVID-19, chloroquine, hydroxychloroquine, remdesivir, cardiovascular burden, review

## Abstract

**Background:**

The world is witnessing an unprecedented crisis with Coronavirus disease 2019 (COVID-19). It is important to accurately analyze the available evidence to provide correct clinical guidance for optimal patient care. We aim to discuss current clinical evidence regarding chloroquine, hydroxychloroquine, azithromycin, remdesivir, and the cardiovascular burden of COVID-19.

**Methods:**

A literature review was performed using PubMed and Google Scholar. Additional clinical trials were identified through the “TrialsTracker” project.

**Results:**

We found conflicting evidence of chloroquine, hydroxychloroquine plus azithromycin, and remdesivir in COVID-19 despite promising early reports of in vitro antiviral activity against severe acute respiratory syndrome coronavirus 2. Some of the current studies have demonstrated adverse drug reactions to chloroquine and hydroxychloroquine + azithromycin. Widespread systemic inflammation and procoagulant/hypercoagulable state, including thrombotic microangiopathy, endothelial dysfunction, bleeding disorder, and thrombosis are increasingly being witnessed in COVID-19. Evidence of cardiac injury and stroke is mostly reported in hospitalized patients; however, large specialized studies that focus on cardiac or neuropathology are lacking.

**Discussion:**

There is no convincing clinical evidence of chloroquine, hydroxychloroquine with or without azithromycin, and remdesivir use in COVID-19. As evidence of systemic inflammation is rapidly unfolding, there is a dire need to maximize our resources to find the best possible solutions to the current crisis while conclusive evidence from clinical trials emerges.

## Introduction

The coronavirus disease 2019 (COVID-19) has rapidly spread across the globe after the detection of the severe acute respiratory syndrome coronavirus 2 (SARS-CoV-2) in December 2019 in Wuhan, China.^1^ The World Health Organization (WHO) declared COVID-19 a pandemic on 11 March 2020, and even after a month, the global community is struggling to contain the virus.^2^ The desperate search for the “active pharmaceutical agents” against SARS CoV 2 is going on,^3^ and as of 07 April 2020, more than 800 clinical trials on COVID-19 can be traced on the “TrialsTracker” project,^4^ which includes nearly 350 intervention trials. As real-world evidence plays a significant role in driving the management protocol during an evolving pandemic, we aim to discuss current clinical evidence regarding chloroquine (CQ), hydroxychloroquine (HCQ), azithromycin, remdesivir, and the cardiovascular burden in COVID-19.

## Methods

A literature review was performed using PubMed and Google Scholar to identify all relevant studies based on our study objective. Nonspecific combinations of the search strings included, (i) coronavirus OR severe acute respiratory syndrome OR 2019-nCoV OR SARS-CoV-2 OR SARS-CoV OR MERS-CoV OR severe acute respiratory syndrome coronavirus 2 OR coronavirus disease OR COVID-19, (ii) quinine OR chloroquine OR CQ OR hydroxychloroquine OR HCQ OR azithromycin OR remdesivir, and (iii) cardiac injury OR myocardial injury OR cardiac pathology OR cardiovascular burden OR cardiovascular disease OR vascular complications OR vascular pathology OR vasculitis OR endothelial dysfunction. Independent analysis of all the studies was done by both the authors due to the paucity of the available randomized clinical trials (RCTs). Additional clinical trials were identified through the “TrialsTracker” project.^4^

### Chloroquine, hydroxychloroquine, and azithromycin

The HCQ, hydroxyl analog of CQ, garnered much attention after the White House press briefing on 19 March 2020.^5^ CQ/HCQ is a recognized drug in malaria and autoimmune diseases. The first antiviral activity of CQ was demonstrated by Tsai et al.^6^ in both avian reticuloendotheliosis virus (REV-A) and human immunodeficiency virus (HIV-1) in the late 1990s. CQ was shown to inhibit the viral surface glycoproteins with a decrement in the viral load. Sperber et al.,^7^ Chiang et al.,^8^ and Boelaert et al.^9^ subsequently consolidated the antiviral activity of the CQ by demonstrating the CQ/HCQ induced inhibition of HIV-1 replication in both T cells and monocytes, thus opening the frontiers of CQ/HCQ combination with other antiviral drugs.^10,11^

Vincent et al.^12^ in 2005 demonstrated the inhibition of SARS CoV in cell culture after treatment with CQ. Savarino et al.^13^ proposed the clinical utility of CQ in SARS in 2003 and famously stated in 2006^14^ that “the broad-spectrum antiviral effects of chloroquine deserve particular attention in a time in which the world is threatened by the possibility of a new influenza pandemic, and the availability of effective drugs would be fundamental during the evaluation of an effective vaccine.”

As SARS-CoV-2 shares genetic and pathological similarity with severe acute respiratory syndrome coronavirus (SARS-CoV) and Middle East respiratory syndrome coronavirus (MERS-CoV), the use of CQ/HCQ in COVID-19 seems logical.^15,16^ Wang et al.^17^ on 04 February 2020, in a letter to the editor in “Nature®” mentioned the effective in vitro combination of remdesivir and remdesivir and chloroquine (CQ) against 2019-nCoV in Vero E6 cells/ATCC-1586. They showed, on the time-of-addition assay, pharmacological action of CQ on both the entry and postentry stages of SARS-CoV-2 with clinically achievable EC90 (effective concentration for 90% inhibition) of 6.90 μM. Yao et al.^18^ subsequently demonstrated HCQ to be more potent than CQ in SARS-CoV-2. Following the lead, several clinical trials were initiated in March 2020 to test CQ/HCQ in COVID-19 across different hospitals in China.^19^

Current evidence of CQ/HCQ in COVID-19 is non-convincing. A small pilot study by Chen et al.^20^ in Shanghai, China with 30 treatment-naive COVID-19 patients failed to demonstrate a statistical difference in the negative nucleic acid viral throat swab between HCQ (400 mg) and control group (86.7%, *n* = 13 vs. 93.3%, *n* = 14; *p* > 0.05). A non-peer-reviewed French trial,^21^ published on medRxiv®, conducted to evaluate the effectiveness of HCQ (600 mg/day), given within 48 h of the admission in 181 SARS-CoV-2 pneumonia patients, showed a nonsignificant decrease in either the intensive care unit (ICU) transfer or death (20.2%, *n* = 16 vs. 22.1%, *n* = 21, Relative Risk (RR): 0.91, 95% confidence interval [CI]: 0.47–1.80) or death rate alone (2.8%, *n* = 3 vs. 4.6%, *n* = 4, RR: 0.61, 95% CI: 0.13–2.89).

In addition to the HCQ alone, the prospect of HCQ + azithromycin has been widely considered in COVID-19. While azithromycin is well studied in Zika and Ebola viruses,^22–24^ its role in COVID-19 is not yet established.^25^ A small open-label nonrandomized French trial^26^ in COVID-19 with 36 patients (20 HCQ group vs. 16 control), with or without azithromycin, showed higher virological clearance with HCQ alone (70% HCQ vs. 12.5% control, *p* = 0.001) and HCQ + azithromycin (100% HCQ + azithromycin vs. 57.1% HCQ alone, or 12.5% control, *p* < 0.001). However, this study had several limitations, including a small number of patients, a short follow-up period and the absence of safety outcomes.

On the other side, a randomized double-blinded, parallel, phase IIb Brazilian trial (CloroCovid-19 Study; NCT04323527)^27^ conducted to assess the safety and efficacy of two different CQ doses (high vs. low) in hospitalized SARS CoV2 patients, in addition to ceftriaxone and azithromycin, had to be prematurely stopped after high dose CQ resulted in QT prolongation in one-fourth of the patients with resultant 13.5% fatality (95% CI: 6.9–23.0%). This adverse drug reaction resonated with the Food and Drug Administration (FDA)^28^ warning on CQ use, which emphasizes the possibility of QT prolongation, either alone or in combination with azithromycin, thereby, necessitating EKG monitoring, especially in patients with coexisting cardiac diseases.

The most recent clinical evidence of HCQ and azithromycin in COVIS 19 is a preliminary retrospective analysis of 368 hospitalized COVID-19 patients in the US,^29^ published in medRxiv®, which is yet to be peer-reviewed. It has shown higher death rates with HCQ alone (27.8%) when compared with HCQ + azithromycin (22.1%) and no HCQ (11.4%). In this study, the risk of death, when compared with the “no HCQ” groups, was higher in both HCQ groups (adjusted HR: 2.61; 95% CI: 1.10–6.17) and HCQ with azithromycin groups (adjusted HR: 1.14; 95% CI: 0.56–2.32). The other primary end point, ventilator rates, were higher in the HCQ groups (13.3%) compared to HCQ and azithromycin (6.9%) and no HCQ groups (14.1%); however, risk of ventilation in all three groups was identical. In this study, HCQ and HCQ + azithromycin were administered late in the clinical course after the patients were intubated.

The latest on this series is a study by Mehra et al.,^30^ which was published on the “Lancet” on 22 May 2020. It was a multinational registry analysis, which included 671 hospitals with 96 032 COVID-19 positive patients. The authors concluded that the CQ/HCQ and macrolide were associated with reduced survival and adverse cardiac outcomes without an apparent benefit. However, the paper was retracted on 5 June 2020^31^ by three of the four authors, including the primary author, citing the concerns regarding data integrity and analytical validity.

While the wait is ongoing, there is no definitive clinical evidence to support the use of CQ/HCQ and/or azithromycin in COVID-19. For now, we can expect more of these conflicting results, until a definitive answer is obtained from a well-designed large-scale RCT. There are around 80 clinical trials listed in “TrialsTracker” related to CQ/HCQ either alone or in combination with other pharmacological agents.^4^ Some of these large clinical trials worth waiting are Trial of Treatments for COVID-19 in Hospitalized Adults (DisCoVeRy; NCT04315948), Anti-Coronavirus Therapies to Prevent Progression of Coronavirus Disease 2019 (ACT COVID19; NCT04324463), Post-exposure Prophylaxis/Preemptive Therapy for SARS-Coronavirus-2 (COVID-19 PEP; NCT04308668), and Hydroxychloroquine Post Exposure Prophylaxis for Coronavirus Disease (COVID-19) (NCT04318444).

### The rising star of ramedesivir

Remdesivir (GS-5734) is a prodrug of 1′-cyano substituted adenine nucleoside analog GS-441524 (Nuc). It was effective in vitro against various viruses^32^ and in vivo in primate–animal models, like rhesus monkeys^33,34^ and African green monkeys.^35^ Remdesivir is well studied in MERS-CoV and SARS-CoV.^36–38^ Sheahan et al.^39^ demonstrated the remdesivir induced in vitro inhibition of SARS/MERS-CoV multiplication in primary epithelial cell cultures of the human respiratory system. This susceptibility to the coronavirus family was directly associated with viral polymerase.^38^

Remdesivir was safely used in the first COVID-19 patient in the US^40^ and subsequently followed up with two more patients.^41^ Compassionate use of remdesivir^42^ in a small multicenter study in severe COVID-19 patients (oxygen saturation 94% or below for 10 days), from 25 January 2020, through 7 March 2020, demonstrated 68% improvement in oxygen support and 57% of the patients came out of ventilatory support following the remdesivir administration. These findings were clinically relevant as the mortality rate among patients receiving invasive ventilation was more than thrice than those without invasive ventilation (18% vs. 5%).

Remdesivir is a relatively new drug and its cardiac toxicity is still unknown. Previously, it was associated with hypotension and bradycardia in Ebola patients.^43^ Some of the early results with remdesivir in COVID-19 are promising, but we cannot establish its clinical efficacy without definitive evidence from the RCTs. Genetic heterogeneity across the Coronaviridae family also limits our previous clinical evidence with remdesivir.^44^ On 27 March 2020, the FDA has authorized emergency use of remdesivir in hospitalized COVID-19 patients through the Emergency Use Authorization (EUA).^45^ However, general use has not been approved and it will be interesting to have the results of the two large prospective clinical trials before we reach a definitive consensus. These two clinical trials are Study to Evaluate the Safety and Antiviral Activity of Remdesivir (GS-5734™) in Participants With Severe Coronavirus Disease (COVID-19) (NCT04292899, phase 3, 6000 estimated patients) and Study to Evaluate the Safety and Antiviral Activity of Remdesivir (GS-5734™) in Participants With Moderate Coronavirus Disease (COVID-19) Compared to Standard of Care Treatment (NCT04292730, phase 3, 1600 estimated patients).

### Cardiovascular complications

The majority of the initial study in COVID-19 exclusively focused on respiratory pathology,^46^ as increased mortality in COVID-19, was mostly attributed to the rapidly developing acute respiratory distress syndrome (ARDS). One of the few initial pathological case reports published in Lancet by Xu et al.^47^ showed only “a few interstitial mononuclear inflammatory infiltrate, but no other substantial damage” in the biopsy samples from heart tissues. However, cardiac injuries in seasonal influenza,^48^ SARS,^49–51^ and MERS,^52^ which are close relatives of COVID-19, are well established. Therefore, it is no coincidence that there will be mounting evidence of cardiac injury, as many mysteries of COVID-19 starts to unfold.^53–55^

Huang et al.^54^ demonstrated myocardial injury in 5 amongst 41 COVID-19 patients and 4 of them required intensive care. Wang et al.^56^ showed acute cardiac injury, shock, and arrhythmia in 7.2%, 8.7%, 16.7%, respectively, among 138 hospitalized COVID-19 patients, most of them requiring ICU admission. A single-center study by Shi et al.^57^ in Wuhan, China, with 416 hospitalized COVID-19 patients reported cardiac injury in 19.7% of the admitted patients. More patients with cardiac injury needed mechanical ventilation (noninvasive: 46.3% vs. 3.9%, *p *<* *.001 and invasive: 22.0% vs. 4.2%, *p *<* *.001), including a higher risk of deaths from both onset of symptoms (HR: 4.26, 95% CI: 1.92–9.49) and admission (HR: 3.41, 95% CI: 1.62–7.16). An identical study by Guo et al.^58^ in 144 COVID-19 patients reported 27.8% of patients with cardiac injury. Mortality in patients was higher in those with elevated troponin alone (37.50%) or with existing cardiovascular disease (CVD) as well as increased troponin (69.44%) when compared with the ones without CVD and normal troponin (7.62%). In most of these studies, increasing age and association of chronic clinical comorbidities were directly associated with a higher likelihood of developing cardiac injury.

Although large specialized studies that focus on cardiac pathology are lacking, smaller case-based study has shown that SARS CoV 2 virus can cause direct endothelial injury and electrolyte imbalance, likely hypokalemia, through angiotensin-converting enzyme 2 (ACE2) receptors, leading to fatal arrhythmia and cardiometabolic compromise.^59,60^ Similarly, treatment of hypertension with the renin–angiotensin–aldosterone system (RAAS) inhibitors can upregulate the tissue expression of the ACE2 receptors and the likelihood of viral transmission or severe disease increases. However, Reynolds et al.^61^ did not find a higher chance of being COVID-19 positive or elevated risk of severe illness in the COVID-19 positive patients based on the use of the common antihypertensive drugs (ACE inhibitors/ARBs, beta-blockers, calcium-channel blockers, and thiazide diuretics). Nevertheless, cytotoxic drugs, like most antiviral drugs, can be associated with drug-related cardiovascular toxicities.^62^ Furthermore, as we are non-hesitant in compassionately using different pharmaceutical agents in search of panacea to COVID-19, the chances of systemic and cardiovascular toxicity cannot be ignored.^63^

### Vascular complications

Widespread systemic inflammation and procoagulant/hypercoagulable state are likely in viral infection.^59,64^ Thrombotic microangiopathy and resultant ARDS and respiratory failure have been reported.^65,66^

Magro et al.^46^ described the complement-induced small vessel injury in the lung and skin of five COVID-19 cases. Similarly, Varga et al.^67^ reported endothelial infection and diffuse endothelial inflammation in COVID-19 patients. The authors attributed the endothelial dysfunction and resultant apoptosis and pyroptosis to either immune-mediated insult or a direct consequence of a viral infection. As ACE2 receptors are located in endothelial cells, it is beyond coincidence that viruses can directly interfere with the vascular system, causing microvascular dysfunction, vasoconstriction, thrombosis, and organ ischemia.

Menter et al.^68^ published autopsy findings of the 21 COVID-19 patients in Switzerland to study the extent of the respiratory system involvement and histopathologic changes in the lungs. The primary cause of death in the postmortem report was respiratory failure with widespread exudative alveolar damage and capillary congestion, which was frequently associated with microthrombi. These findings were present in the patients despite the commencement of the anticoagulation therapy. Furthermore, four patients had pulmonary embolism (PE), three alveolar hemorrhages, other three generalized thrombotic microangiopathies, and one vasculitis. This study purported the viral-induced vascular disruption as part of the COVID-19 advancement.

At present, numerous studies have reported pro-coagulation state and a higher risk of deep vein thrombosis (DVT), venous thromboembolism (VTE), and disseminated intravascular coagulation (DIC) in COVID-19.^69–72^ Thrombotic complications are more commonly seen in critically ill patients. Klok et al.^73^ reported thrombotic complications in almost one-third of the seriously ill ICU COVID-19 patients.

There are reports of PE in patients even without visible VTE risk.^70^ An autopsy of the first 12 COVID-19 deaths in a single center in Germany^74^ showed 58% had DVT and these patients had no clinical signs or symptoms of DVT before death. Similarly, the PE was attributed to the cause of death in one-third of the patients. A recently updated follow-up Dutch study COVID-19 ICU patients by Klok et al.^75^ reported PE in 87% of the patients (*n* = 65/75) among those with thrombotic events and showed thrombotic complications was linked with significantly higher (more than five times) risk of all-cause death. Al-Ani et al.^76^ performed a pooled analysis of the reported studies with 1765 patients and indicated VTE in approximately 20% of the patients, with more than double (49%) cumulative incidence of the VTE during hospitalization. This study concluded that the VTE is an important complication in COVID-19, especially in critically ill patients admitted in ICU. Although this study had high statistical heterogeneity, it provides us a preliminary ground to acknowledge VTE as a possible complication in critically ill COVID-19 patients.

There are compelling clinical evidence of DIC in COVID-19 positive deaths. A higher proportion of in-hospital deaths in COVID-19 satisfied the diagnostic criteria for DIC in a study by Tang et al.^77^ compared to the survivors (71.4% vs. 0.6%). Ai et al.^78^ showed similar results with higher DIC in non-survivor during follow-up compared to the survivor (71% vs. 0.6%, *p* < 0.001). The definition of the DIC on these two studies was based on the International Society on Thrombosis and Hemostasis (ISTH) diagnostic criteria.^79^ Deng et al.^80^ reported even higher DIC incidence among non-survivors (6.4% vs. 0, *p* = 0.006), but the study did not specify the DIC diagnostic criteria. Notably, the DIC in COVID-19 is considered to be different from the DIC seen in sepsis as it is associated with decreased platelet count, increased prothrombin time, and subsequent bleeding tendency.^72,76^

It is necessary to understand the pathological process of SARS-CoV 2 in humans to decode its clinical course. Studies have shown poor prognosis in COVID-19 patients with abnormal coagulation parameters,^77^ and many critically ill patients have subsequently benefitted with anticoagulation therapy.^81^ Hypercoagulable states can predispose patients to acute coronary syndrome^82^ or stroke,^83,84^ strengthening the need for thrombotic prophylaxis in COVID-19. For now, it does seem plausible that severe acute infection with SARS-CoV 2 with cytokine excess could overload the myocardial demand, disrupt plaque stability, and increases thrombotic complications, especially in patients with chronic comorbidities and/or underlying CVD, resulting in high morbidity and mortality.^59,85,86^

### Recommendations

We are already overburdened with a staggering amount of clinical data with conflicting evidence in COVID-19. These available pieces of evidence must be analyzed accurately to provide correct clinical evidence for optimal patient care. As there is a dire need to maximize our resources to find the best possible solutions to the current crisis, we firmly believe that machine learning (ML) and artificial intelligence (AI) should be utilized in analyzing these big data. Harnessing AI, big data analysis, and bioinformatics will allow us to scrutinize how we can provide the best option to our patients, making it possible to create a bioinformatics modeling that can surpass RCT for a conclusive answer to many unanswered questions. Without delay, adaptive platform designs must be structured to promote maximum learning across the world to adjust how we deliver the best care to our patients.

## Conclusion

There is no convincing clinical evidence of CQ, HCQ, with or without azithromycin, and remdesivir in COVID-19. Unfortunately, we might have to wait for months or even years before we have definitive results from the ongoing RCTs. The cardiovascular burden due to the direct consequence of the SARS CoV 2 and compassionate use of the cytotoxic drugs are also progressively emerging. Therefore, it is crucial to follow the standard preventive and management guidelines to avoid clinical burden in COVID-19.
